# Oral Collagen Drink for Antiaging: Antioxidation, Facilitation of the Increase of Collagen Synthesis, and Improvement of Protein Folding and DNA Repair in Human Skin Fibroblasts

**DOI:** 10.1155/2020/8031795

**Published:** 2020-05-11

**Authors:** Ping Lin, Nan Hua, Yu-Chen Hsu, Kai-Wen Kan, Jia-Haur Chen, Yung-Hao Lin, Yung-Hsiang Lin, Chen-Meng Kuan

**Affiliations:** ^1^Research & Design Center, TCI Co., Ltd., Taipei 114, Taiwan; ^2^Zhejiang Kazman Biotechnology Co., Ltd., Xiaoshan District 311200, China; ^3^Research & Design Center, TCI Gene Inc., Taipei 114, Taiwan; ^4^Global Business Center, TCI Co., Ltd., Taipei 114, Taiwan

## Abstract

This work unveils a fish collagen drink for improvement of skin aging. Previous studies frequently discussed the skin aging from the angle of the representative characteristics of collagen loss and the oxidative-induced expression of proteolytic enzymes matrix metalloproteinases (MMPs), but few groups comprehensively investigated the efficacy of oral hydrolyzed collagen for enhancing protein folding and DNA repair as well as improving notable cell behaviors. To delineate the broad perspective on delaying skin aging, we inspected the collagen drink-treated fibroblast cells from the molecular and cellular aspects. The results show that the collagen drink could perform the compact antiaging effects on ROS inhibition, the facilitation of the synthesis of extracellular matrix (ECM) proteins, the increase of mitochondrial activity, and improvement of the gene expression regarding correct protein folding, DNA mismatch repair (MMR) and base excision repair (BER). Although the experimental results are built on the cellular models, we believe that the positive outcomes can provide more details on the influence of oral hydrolyzed collagen supplement for antiaging. In short, we have successfully proved that the synergistic effect of the collagen drink could not only reduce the oxidative damage but also ameliorate the cell functionality to compensate the harmful effects induced by UVA.

## 1. Introduction

Collagen accounts for around 30% of the protein composition of the human body and is a predominant protein in the connective tissues, i.e., skin, blood vessel, and cartilage [1]. Collagen, coupled with elastin, hyaluronate (HA), and proteoglycans, forms ECM, which endows cells with mechanical characteristics and molecule cues for either cell stabilization or functionalization [2]. Unlike other organs, skin directly exposes to the environment, and its appearance represents the epitome of the overall aging process [3]. Skin aging is generally characterized by wrinkles, laxity, brown spots, thickening, and coarseness, and these deteriorations are associated with the degradation of ECM proteins and the accumulation of advanced glycation end products (AGEs) [4, 5]. Collagen types I and III participate in around 95% of the skin constitution and are responsible for the resilience, strength, durability, and elasticity in the skin property [1, 6]. With the passage of time, aging elicits the reduction of 1% of collagen content yearly [7]. Chronological aging is mainly contributed by the attenuation of collagen and elastin synthesis, while extrinsic aging is subject to disorganized collagen fibers and the accumulation of haphazard elastin fragmentation [8]. Several studies have unveils that old skin manifests the low expression levels of type I procollagen and upregulation of the collagenases matrix metalloproteinases (MMPs) in comparison with young skin [9, 10]. Extrinsic aging is often related to UV irradiation, inappropriate lifestyles, and environmental pollutants [11]. Of these deleterious factors, UV irradiation, including UVA (314-400 nm), UVB (290-320 nm), and UVC (100-280 nm), is the primary cause to premature skin aging—photoaging—and leads to direct DNA damage or UV-induced oxidative damages to macromocules (i.e., lipid, protein, and DNA) [12]. For instance, reactive oxygen species (ROS) may attack DNA base deoxyguanosine and yield the mutagenesis of 8-oxoguanine and GC-TA transversion mutation [13]. It has been shown that the ROS levels were highly increased in human skin after the 15-minute exposure to UV irradiation [14]. The UV-mediated ROS may activate the signaling pathways correlated with the nuclear transcription factors of AP-1 and nuclear factor kappa-light-chain-enhancer of activated B (NF-*κ*B) [15]. AP-1 and NF-*κ*B cascades enhance the expression of MMP genes (e.g., MMP-1, MMP-3) and inhibit collagen synthesis [16]. Moreover, NF-*κ*B can stimulate the production of inflammatory cytokines (e.g., tumor necrosis factor-*α* (TNF-*α*), interleukin (IL)-1, IL-6, and IL-8) as well as adhesion molecules (e.g., intercellular adhesion molecule-1 (ICAM-1)) [17].

Oral supplementation of hydrolyzed collagen is beneficial for stimulating the formation of ECM proteins, slowing UV-induced aging, and improving fibroblast proliferation [18–20]. Hydrolyzed collagen, small peptides with the lower molecular weights (0.3-8 kDa), is easy to be absorbed by the intestine and available for tissues [21]. The underlying mechanisms for the facilitation of collagen synthesis can be divided into two aspects: (i) the absorbed amino acids after the digestion of peptides act as building materials for collagen production in fibroblasts or (ii) the oligopeptides as ligands adhere on the surface of fibroblasts and stimulate the secretion of collagen, elastin, and HA [1]. In addition to the collagen source of land animals, fish collagen has recently been highlighted in several applications in light of the availability of the fish waste (e.g., bone and scale) and its unique amino peptides [22]. Enormous cellular, animal, and clinical studies have confirmed that hydrolyzed fish collagen is able to combat the process of skin aging through boosting the efficiency of collagen synthesis, inhibiting MMPs expression, and/or improving skin indexes (such as hydration, wrinkles, *etc.*) [21–24]. However, to the best of our knowledge, few reports mentioned the comprehensive view of antiaging regarding ROS inhibition, the facilitation of collagen and elastin production, and, most importantly, improvement in protein folding and DNA repair for a hydrolyzed collagen product. Here, this work demonstrates the relatively compact *in vitro* analyses on these indexes as to better understanding the benefits of fish collagen in skin aging.

## 2. Materials and Methods

### 2.1. Materials and Instrument

Elastin Collagen Peptide Complex Juice Drink (YAMII, Zhejiang Kazman Biotechnology Co., China; ingredients: water, fish collagen peptide powder (12%), banana powder, apple essence, cherry essence, *γ*-aminobutyric acid, grape powder, alma powder, acai powder, perilla seed powder, elderberry powder, brown rice powder, honey, vitamin C, erythritol, citric acid, fructose syrup, sucrose, flavor), human skin fibroblast (CCD-966Sk; ATCC®, CRL-1881), minimum essential medium with 10% FBS, 1% penicillin/streptomycin, and 1 mM sodium pyruvate (Gibco), 3-(4,5-dimethylthiazol-2-yl)-2,5-diphenyltetrazolium bromide (MTT, Amresco), phosphate-buffered saline (PBS, Gibco), DMSO (ECHO), collagen assay kit (Sircol), mitochondrial membrane potential detection kit (BD), 2′,7′-dichlorofluorescin diacetate (DCFH-DA, Sigma-Aldrich), elastin assay kit (Biocolor), RNA extraction kit (Genaid Biotech), nCounter® platform (NanoString Technologies), flow cytometry (BD) ELISA reader (BioTek).

### 2.2. Cell Viability Assay

5 × 10^3^ CCD-966Sk cells in 200 *μ*L culture media were dispersed into each well in a 96-well plate and incubated at 37°C for 24 hours. The media were replaced with fresh ones with 0%, 0.125%, 0.25%, or 0.5% collagen drink. After 24 hours incubation, the cells were irradiated under UVA (10 J/cm^2^). Note that the UVA dosage was referenced by other associated studies [25]. Subsequently, 15 *μ*L MTT (4 mg/mL) was added to each well for 4 hours reaction. Afterwards, the solution was removed, and 50 *μ*L DMSO was added into each well to solve formazan crystal in 10 minutes. The absorption results (at 570 nm) were recorded by an ELISA reader.

### 2.3. Collagen Assay

2 × 10^4^ CCD-966Sk cells in 0.5 mL culture media were added to each well in a 24-well plate followed by 24 hours incubation. Then, the media were replaced with 0%, 0.125%, 0.25%, or 0.5% collagen drink in the media without FBS followed by the UVA (10 J/cm^2^) treatment. Following 48 hours incubation, the cells were collected from the well for further analysis of collagen content by the collagen assay.

### 2.4. Mitochondrial Membrane Potential

1 × 10^5^ cellsCCD-966Sk cells in 2 mL culture media were added to each well in a 6-well plate followed by 24 hours incubation. Then, the media were replaced with 0%, 0.125%, 0.25%, or 0.5% collagen drink prepared in the media. Following 24 hours incubation, the media were removed, and the cells were rinsed with PBS twice. Afterwards, the cells were collected in a 1.5 mL microcentrifuge tube after trypsin digestion and underwent centrifugation. The supernatant was discarded, and 100 *μ*L JC-1 dye solutions (dye preparation as referenced by the kit protocol) were pipetting in the tube and interacted with the cells in 15 minutes. In the end, the dye solutions were removed, and the cells were resuspended in 500 *μ*L PBS with 2% FBS. Finally, the cells were loaded into a flow cytometry for fluorescence analysis.

### 2.5. ROS Assay

1 × 10^5^ CCD-966Sk cells in 2 mL media were added to each well in a 6-well plate and underwent 24 hours incubation. Then, the media were replaced with the 0%, 0.25%, or 0.5% collagen drink, and they were incubated for 1 hour. Afterwards, 10 *μ*g/mL of DCFH-DA (a ROS indicator) solutions were added to each well and waiting for 15 minutes. Then, the cells were irradiated under 10 J/cm^2^ UVA irradiation (10 J/cm^2^). In the end, the cells were collected by the use of trypsin, and the suspending cells were loaded into a cytometry (Ex: 450-490 nm; Em: 510-550 nm).

### 2.6. Elastin Assay

1 × 10^5^ CCD-966Sk cells in 2 mL media were dispersed in each well in 6-well plates and were incubated for 24 hours. Then, the media were replaced with the fresh media with 0%, 0.125%, 0.25%, or 0.5% collagen drink followed by the UVA (10 J/cm^2^) treatment. After 48 hours incubation, cells were collected by the use of trypsin, and the elastin levels of the cells were analyzed by the elastin assay kit.

### 2.7. Analysis of mRNA Expression

1.5 × 10^5^ CCD-966Sk cells (treated with/without 10 J/cm^2^ UVA) in 2 mL media with 0.25% or 0.5% collagen drink were dispersed into each well in 6-well plates and were incubated for 24 hours. Then, the analysis of the mRNA expression level was conducted by an nCounter® platform, and the operation was following the recommendations of the protocol.

### 2.8. Statistical Analysis

All the measurement results were analyzed by analysis of variance (ANOVA) and *post hoc* test in GraphPad Prism; *p* < 0.05 was considered a significant difference.

## 3. Results and Discussion

### 3.1. Cell Viability

**Figure 1:**
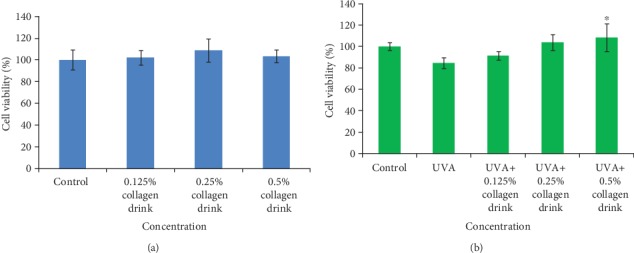
Result of cell viability of fibroblasts. (a) CCD-966Sk cells were treated with 0%, 0.125%, 0.25%, or 0.5% for 24 hrs. (b) CCD-966Sk cells were treated with 0.125%, 0.25%, or 0.5% for 24 hrs followed by UVA treatment (10 J/cm^2^). (*n* = 3, mean value ± S.D.) (corresponding to the UVA group: ∗*p* < 0.05).

Before the testing, we prepared 0.125%, 0.25%, and 0.5% of collagen drinks and attempted to verify their isotonicity to fibroblasts (Figure S1). We did not discover any detrimental effect of osmotic pressure on CCD-966Sk cells from the drinks as evidence by the morphological comparison among the control and experimental groups.  Figure 1 shows the cell viability results of fibroblasts after treatment with different concentrations of collagen drinks (with/without UVA irradiation). 0.125%, 0.25%, and 0.5% of collagen drinks slightly enhanced the cell proliferation capability in comparison with the control group and caused no harmful effects on the cell growth (Figure 1(a)). In addition, UVA treatment was introduced to the study (after the sample treatment) to assess the influence of collagen drink and the UVA effect. We employed UVA rather than UVB as a photoaging-induced source given that some studies have stressed that UVA can penetrate deeper than UVB into the skin (UVA: 20-30% to dermis; UVB: 10% to dermis) and is more appropriate for investigating the profound photoaging process in skin [26]. 0.125%, 0.25%, and 0.5% of collagen drinks could increase the cell viability levels of UVA-treated fibroblasts by 7.9%, 22.3%, and 28.1%, respectively, as compared with the UVA group (Figure 1(b)). The improvement exhibited a dose-dependent effect, but only the highest concentration showed significant improvement. Also, we were curious about the outcomes of fibroblasts treated with UVA before the step of collagen drink treatment, which somewhat resembles the cell repair process, and ones simultaneously treated with UVA and collagen drink (Figures S2). While fibroblasts were treated with UVA ahead of sample treatment, the improvement of cell viability was inversely correlated with the increasing concentration of the collagen drink (Figures S2A). The consequence might be attributed to the fact that its high-level content hindered the efficiency of cell repair and, in turn, impaired the proliferation ability. On the other hand, the cotreatment result was similar with Figure 1(b) (Figures S2A). This might be due to the absorbance of the solutes in the collagen drink.

**Figure 2:**
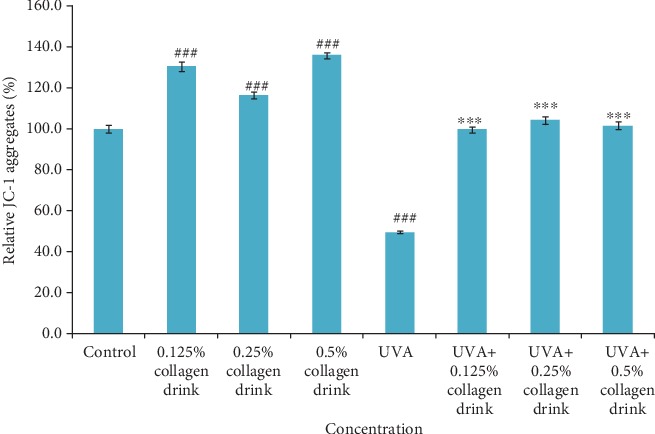
Result of mitochondrial activities of fibroblasts. CCD-966Sk cells were treated with 0%, 0.125%, 0.25%, or 0.5% for 24 hrs followed by UVA treatment (10 J/cm^2^) for the UVA-related groups. (*n* = 3, mean value ± S.D.) (corresponding to the control group: ###*p* < 0.001; corresponding to the UVA group: ∗∗∗*p* < 0.001).

Moreover, we utilized a mitochondrial assay to look into the detailed cellular activity in fibroblasts after treatment with collagen drink and/or UVA (Figures 2, S3). In comparison with the control group, 0.125%, 0.25%, and 0.5% of collagen drinks could increase the mitochondrial activities of fibroblasts by 30.6%, 16.4%, and 36.1%, respectively. Regarding photoaging improvement, the mitochondrial activities of the UVA-treated cells were improved by 100.9%, 110.2%, and 105.4%, respectively, followed by treatment with corresponding 0.125%, 0.25%, and 0.5% of collagen drinks. Hence, the collagen drinks remarkably boost the mitochondrial membrane potential in original and UVA-treated fibroblasts. On the basis of these results, the collagen drink might substantially improve the cell viability and galvanize the mitochondrial activity of fibroblasts.

### 3.2. Collagen and Elastin Synthesis

**Figure 3:**
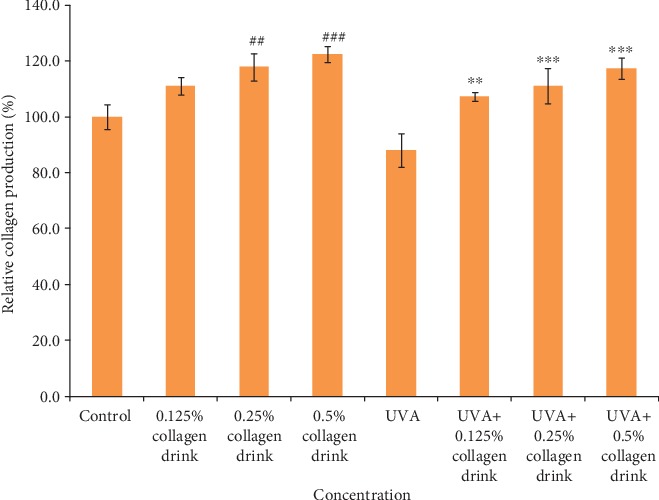
Result of collagen production levels of fibroblasts. CCD-966Sk cells were treated with 0%, 0.125%, 0.25%, or 0.5% for 48 hrs followed by UVA treatment (10 J/cm^2^) for the UVA-related groups. (*n* = 3, mean value ± S.D.) (corresponding to the control group: ##*p* < 0.01; ###*p* < 0.001; corresponding to the UVA group: ∗∗*p* < 0.01; ∗∗∗*p* < 0.001).

**Figure 4:**
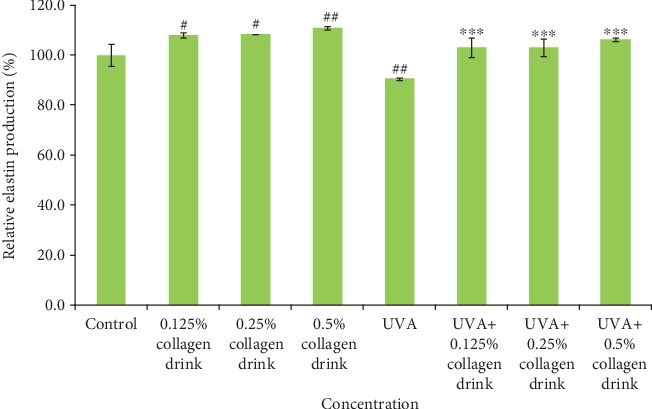
Result of elastin production levels of fibroblasts. CCD-966Sk cells were treated with 0%, 0.125%, 0.25%, or 0.5% for 48 hrs followed by UVA treatment (10 J/cm^2^) for the UVA-related groups. (*n* = 3, mean value ± S.D.) (corresponding to the control group: ##*p* < 0.01; ###*p* < 0.001; corresponding to the UVA group: ∗∗*p* < 0.01; ∗∗∗*p* < 0.001).

We further assessed the effect of the facilitation of collagen and elastin production in fibroblasts as they were treated with the different concentrations of collagen drinks for 48 hrs. The incubation setting allowed distinctive observation on cellular collagen and elastin secretion in CCD-966Sk cells. 0.125%, 0.25%, and 0.5% of collagen drinks could improve the relative collagen production levels of fibroblasts by 11.1%, 17.9%, and 22.5%, respectively, as compared with the control group; 0.125%, 0.25%, and 0.5% of collagen drinks could improve the relative collagen production levels of UVA-treated fibroblasts by 17.9%, 20.7%, and 24.9%, respectively, in comparison with the UVA group (Figure 3). The results indicate that the collagen drink could substantially and significantly improve the ability of collagen synthesis in fibroblasts without the concern of impairment of cell viability after 48-hour sample treatment. Figure S4 indicates the similarity with the results of the cells after 24-hour sample treatment (Figure 2)—a sudden drop trend of relative cell viability did not appear. The improvement effect complies with a dose-dependent tendency, which can be explained by the theory of the better efficiency of collagen synthesis benefiting from high levels of exogenous proteins [27]. Apart from the encouraging result of collagen production, the collagen drink also positively enhanced the elastin production of fibroblasts. 0.125%, 0.25%, and 0.5% of collagen drinks increased the relative elastin production levels of fibroblasts by 7.9%, 8.4%, and 10.9%, respectively, as compared with the control group; 0.125%, 0.25% and 0.5% of collagen drinks could improve the relative collagen production levels in UVA-treated fibroblasts by 13.8%, 13.8%, and 17.6%, respectively, in comparison with the UVA group (Figure 4).

**Figure 5:**
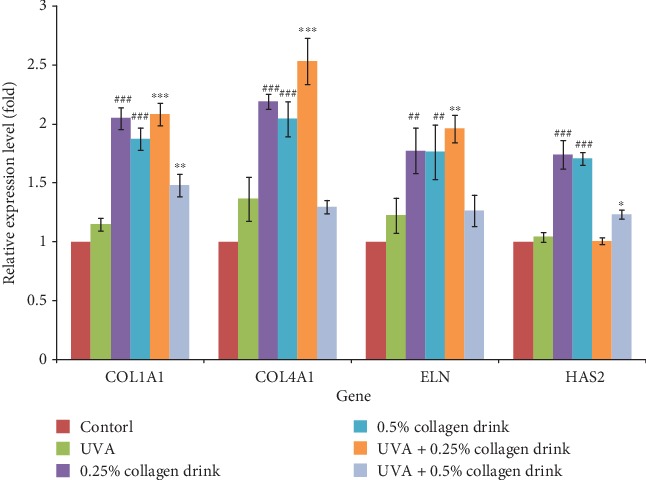
Result of the gene analysis for ECM-associated components. CCD-966Sk cells were treated with 0%, 0.125%, 0.25%, or 0.5% for 24 hrs followed by UVA treatment (10 J/cm^2^) for the UVA-related groups. (*n* = 3, mean value ± S.D.) (corresponding to the control group: ##*p* < 0.01; ###*p* < 0.001; corresponding to the UVA group: ∗*p* < 0.05; ∗∗*p* < 0.01; ∗∗∗*p* < 0.001).

On top of that, we analyzed the gene expression of ECM-associated components (i.e., collagen (COL), elastin (ELN), and hyaluronate (HAS)) (Figure 5). 0.25% and 0.5% of collagen drinks could significantly upregulate the expression of *COL1A1*, *COL4A1*, *ELN*, and *HAS2* genes for original fibroblasts. After UVA treatment, the collagen drink positively modulated these gene expression levels. However, the degrees of improvement for the genes varied with the concentration of collagen drink. In general, 0.25% collagen drink conferred the better upregulation effects on most genes than 0.5% one. The lower gene expression in 0.5% collagen drink group might be due, in part, to some environmental stress issues. Unlike collagen and elastin expression at cellular level, 24-hour sample treatment was enough for clear identification on distinctive gene expression, so we did not adopt 48 hours incubation period in this experiment. Taken together, we have verified that the collagen drink can indeed upregulate the expression of collagen and elastin and their associated genes, which may reinforce the ECM structure and recover the deleterious effect of aging.

### 3.3. UVA-Induced Oxidative Stress

**Figure 6:**
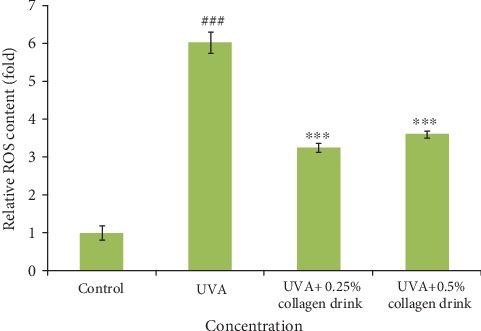
Result of relative ROS content of fibroblasts. CCD-966Sk cells were treated with 0%, 0.25%, or 0.5% for 24 hrs followed by UVA treatment (10 J/cm^2^). (*n* = 3, mean value ± S.D.) (corresponding to the control group: ###*p* < 0.001; corresponding to the UVA group: ∗∗∗*p* < 0.001).

**Figure 7:**
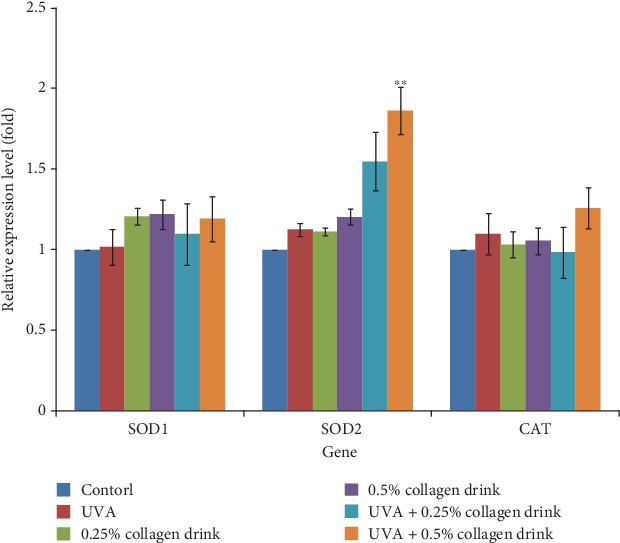
Result of the gene analysis related to anti-oxidative enzymes. CCD-966Sk cells were treated with 0%, 0.25%, or 0.5% for 24 hrs followed by UVA treatment (10 J/cm^2^) for the UVA-related groups. (*n* = 3, mean value ± S.D.) (corresponding to the UVA group: ∗∗*p* < 0.01).

Oxidative stress is a critical driving factor for cell aging. Thus, we attempted to study the antioxidative capacity of the collagen drink under the circumstance of UVA-induced oxidative stress (Figures 6 and 7). Considering the previous experiments, the degrees of improvement were positively correlated with the increasing concentration of collagen drink. The relative ROS levels of 0.25% and 0.5% of collagen drink groups were 3.2 and 3.6 folds, respectively (Figure 6). Compared with the UVA group, the collagen drink could least decrease 2.4-fold UVA-induced oxidative stress. Both concentrations performed the similar inhibition effects on ROS production in fibroblasts.  Figure 7 indicates the result of the expression of *SOD1* (superoxide dismutase1), *SOD2*, *CAT* (catalase) genes. The upregulation of *SOD2* and *CAT* genes of fibroblasts was achieved after treatment with the collagen drink despite positive *CAT* modulation only being observed in 0.5% of collagen drink. The outcome was correlated with the ROS result. For the original cells, 0.25% and 0.5% of collagen drinks could remarkably increase the expression levels of *SOD*1 and *SOD2* genes. Accordingly, the collagen drink can mitigate the influence of UVA-induced oxidative stress.

### 3.4. Protein Folding and DNA Repair

**Figure 8:**
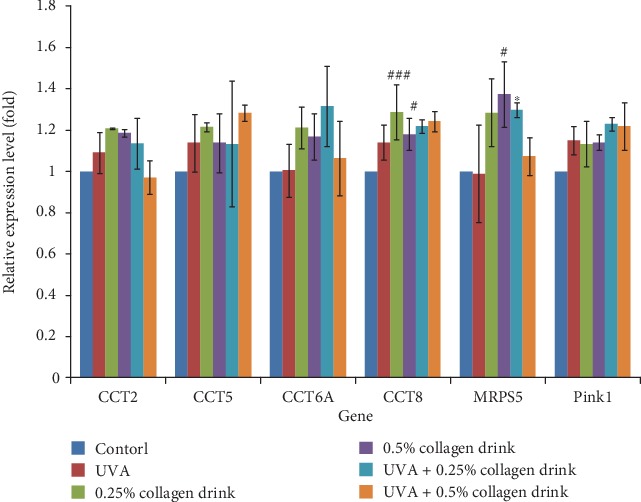
Result of the gene analysis related to protein folding. CCD-966Sk cells were treated with 0%, 0.25%, or 0.5% for 24 hrs followed by UVA treatment (10 J/cm^2^) for the UVA-related groups. (*n* = 3, mean value ± S.D.) (corresponding to the control group: #*p* < 0.05; ###*p* < 0.001; corresponding to the UVA group: ∗*p* < 0.05).

**Figure 9:**
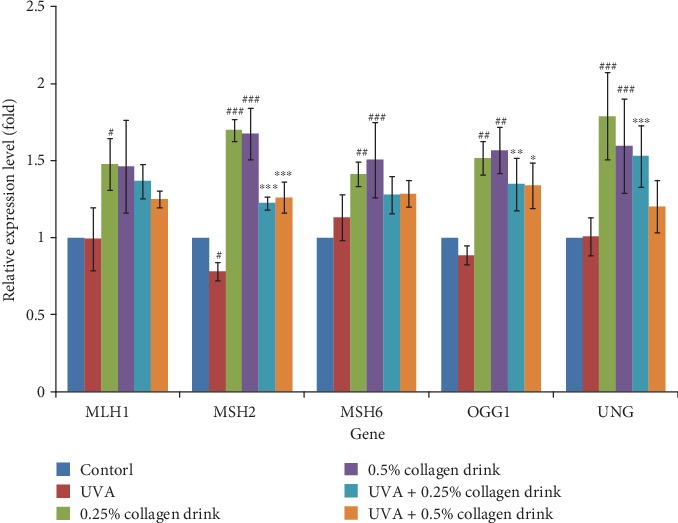
Result of the gene analysis related to MMR and BER. CCD-966Sk cells were treated with 0%, 0.25%, or 0.5% for 24 hrs followed by UVA treatment (10 J/cm^2^) for the UVA-related groups. (*n* = 3, mean value ± S.D.) (corresponding to the control group: #*p* < 0.05; ##*p* < 0.01; ###*p* < 0.001; corresponding to the UVA group: ∗*p* < 0.05; ∗∗∗*p* < 0.001).

A decline of mitochondrial activity is involved in the aging process and the onset of myriad diseases [28]. Conversely, remaining appropriate mitochondrial status is a means to delay aging and lower the risks of certain diseases. We, therefore, investigated the expression of some genes which may affect protein folding, DNA mutation, and DNA repair (Figures 8 and 9). CCT (chaperonin containing TCP1 complex), consisting of CCT1-CCT8 subunits, is a chaperon complex for correct protein folding, and its implementation is associated with cell life span, division, and migration [29]. PTEN-induced kinase 1 (PINK1)/Parkin-mediated mitophagy is recognized as a reliever for mitochondria DNA mutation in flies, and the mutations are associated with the onset of neurodegenerative diseases [30]. *MLH1* (mutL homolog 1), *MSH2* (mutS homolog 2), and *MSH3* are MMR-related genes, whose mutations result in gastrointestinal cancers [31]. Moreover, *OGG1* (8-oxoguanine DNA glycosylase) and *UNG* (uracil DNA glycosylase) genes engage in BER [31, 32].  Figure 8 shows the result of the expression of *CCT2*, *CCT5*, *CCT6A*, *CCT8*, and *PINK1* genes. The collagen drink could obviously enhance the activities of correct protein folding and mitophagy for the original cells, whereas the detrimental complications of UVA irradiation counteracted the improvement effect of the collagen drink.  Figure 9 indicates that the collagen drink could significantly ameliorate the performance of MMR and BER, i.e., the upregulations of *MLH1*, *MSH2*, *MSH6*, *OGG1*, and *UNG* genes, in the original and UVA-treated fibroblasts. In a word, DNA repair efficiency can be improved by the collagen drink.

As a result, the synergistic effect of the collagen drink can enhance cell viability and the expression of collagen and elastin, reduce the ROS damage, and improve the protein folding and DNA repair the risks for lowering the risk of DNA mutation in fibroblasts. Although the experimental results are built on the cellular models and cannot fully reflect the exact benefit of the collagen drink in humans (due to the digestive system), we humbly believe that the positive outcomes still can bring a more meticulous insight into the benefits of oral collagen drink for antiaging. Noticeably, the collagen content (12%) in the collagen drink can perform the real clinical efficacy in accordance with other researches [33].

## 4. Conclusions

This work demonstrates the comprehensive antiaging effect of a fish hydrolyzed collagen drink on ROS inhibition, the facilitation of ECM protein synthesis, and the improvement of protein folding and DNA repair. The results of cell behaviors indicate that the collagen drink could improve cell viability as well as promote collagen and elastin synthesis. In addition, molecular results also indicate that the collagen drink could enhance the gene expression with respect to ECM protein synthesis, antioxidative enzymes, protein folding, DMR, and BER. In a word, the fish collagen drink exerts the synergetic effect on delaying skin aging by interfering with the aging parameters and compensating the oxidative damage and physiological loss in human fibroblasts.

## Data Availability

The authors have confirmed that all the experimental results for the main findings of this work are available in the article and the supplementary materials.

## Supplementary Materials

Supplementary Materials Supplementary Materials Figure 1. Optical images of fibroblasts after treatment with different concentrations of collagen drinks for 24 hours. Figure 2. Result of cell viability of fibroblasts. (A) CCD-966Sk cells were treated with 0%, 0.125%, 0.25%, or 0.5% for 24 hrs following UVA treatment (10 J/cm2). (B) CCD-966Sk cells in 0.125%, 0.25%, or 0.5% collagen drink were treated with UVA (10 J/cm2) followed by 24-hour incubation. (*n* = 3, mean value ± S.D.) (Corresponding to the control group: ##*p* < 0.01; corresponding to the UVA group: ∗∗*p* < 0.01) Figure 3. Results of flow cytometry pattern and fluorescence image of mitochondrial activity in fibroblasts. CCD-966Sk cells were treated with JC-1 dye (red signal) to assess the mitochondrial activity in fibroblasts and their signals were analysed by a flow cytometry by the percentage of cell signal at the P1 zone, which was defined by a ratio of 1(green signal from FITC channel): 1 (red signal from PE-A channel). (A, I) Control. (B, J) 0.125% collagen drink. (C, K) 0.25% collagen drink. (D, L) 0.5% collagen drink. (E, M) UVA only. (F, N) UVA + 0.125% collagen drink. (G, O) UVA + 0.25% collagen drink. (H, P) UVA + 0.5% collagen drink Figure 4. Result of cell viability of fibroblasts. CCD-966Sk cells were treated with 0.125%, 0.25%, or 0.5% for 48 hrs. (*n* = 3, mean value ± S.D.)
