# Supplementary material for: Oral Collagen Drink for Antiaging: Antioxidation, Facilitation of the Increase of Collagen Synthesis, and Improvement of Protein Folding and DNA Repair in Human Skin Fibroblasts
Source: Oxid Med Cell Longev. 2020 May 11;2020:8031795. doi: 10.1155/2020/8031795 (PMC7238338; doi:10.1155/2020/8031795)
Supplement: Supplementary Materials — Figure 1. Optical images of fibroblasts after treatment with different concentrations of collagen drinks for 24 hours. Figure 2. Result of cell viability of fibroblasts. (A) CCD-966Sk cells were treated with 0%, 0.125%, 0.25%, or 0.5% for 24 hrs following UVA treatment (10 J/cm2). (B) CCD-966Sk cells in 0.125%, 0.25%, or 0.5% collagen drink were treated with UVA (10 J/cm2) followed by 24-hour incubation. (n = 3, mean value ± S.D.) (Corresponding to the control group: ##p < 0.01; corresponding to the UVA group: ∗∗p < 0.01) Figure 3. Results of flow cytometry pattern and fluorescence image of mitochondrial activity in fibroblasts. CCD-966Sk cells were treated with JC-1 dye (red signal) to assess the mitochondrial activity in fibroblasts and their signals were analysed by a flow cytometry by the percentage of cell signal at the P1 zone, which was defined by a ratio of 1(green signal from FITC channel): 1 (red signal from PE-A channel). (A, I) Control. (B, J) 0.125% collagen drink. (C, K) 0.25% collagen drink. (D, L) 0.5% collagen drink. (E, M) UVA only. (F, N) UVA + 0.125% collagen drink. (G, O) UVA + 0.25% collagen drink. (H, P) UVA + 0.5% collagen drink Figure 4. Result of cell viability of fibroblasts. CCD-966Sk cells were treated with 0.125%, 0.25%, or 0.5% for 48 hrs. (n = 3, mean value ± S.D.) [file 8031795.f1.pdf]

Supplementary information

Oral collagen drink for anti-aging: anti-oxidation, facilitation of the increase of collagen synthesis, and improvement of protein folding and DNA repair in human skin fibroblasts

Ping Lin[1], Nan Hua[2], Yu-Chen Hsu[3], Kai-Wen Kan[3], Jia-Haur Chen[3], Yung-Hao Lin[4], Yung-Hsiang Lin[1], Chen-Meng Kuan[1]

[1] Research & Design Center, TCI Co., Ltd., Taipei 114, Taiwan
[2] Zhejiang Kazman Biotechnology Co., Ltd., Xiaoshan District 311200, China
[3] Research & Design Center, TCI Gene Inc., Taipei 114, Taiwan
[4] Global Business Center, TCI Co., Ltd., Taipei 114, Taiwan

Correspondence should be addressed to Chen-Meng Kuan; Chenmeng.Kuan@tci-bio.com

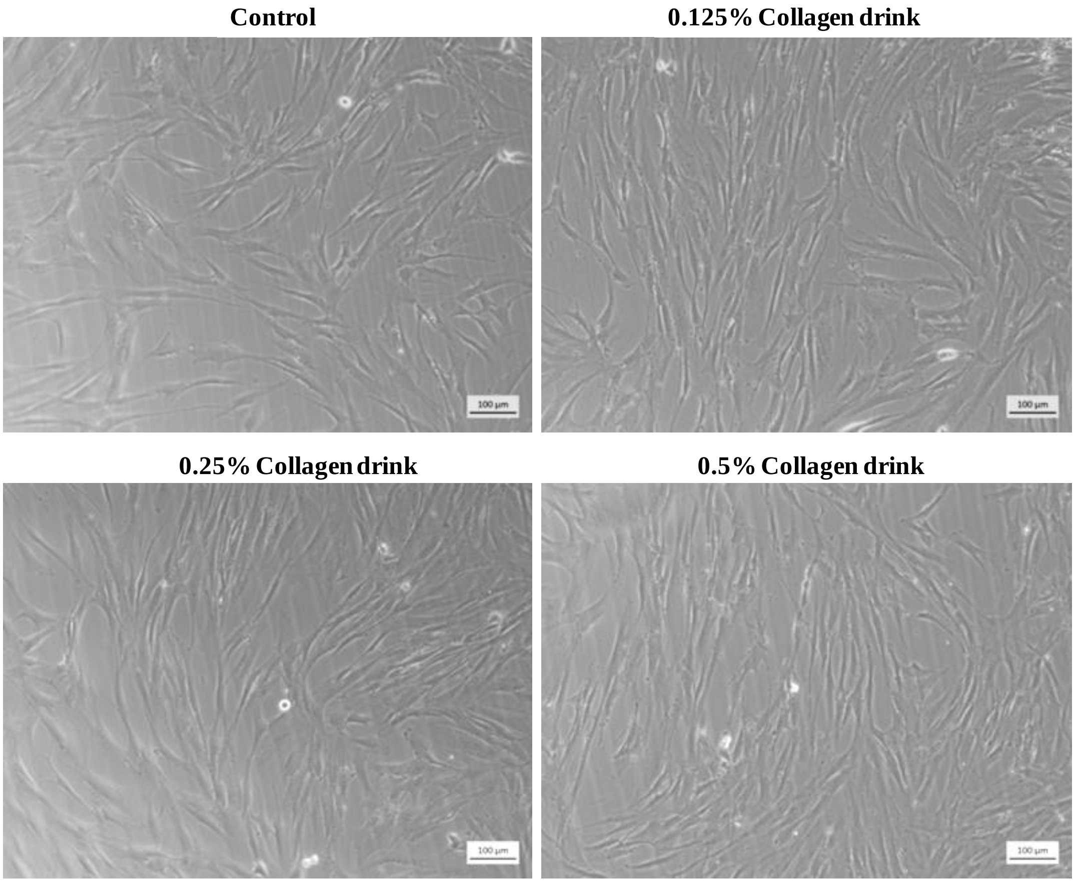

Figure 1. Optical images of fibroblasts after treatment with different concentrations of collagen drinks for 24 hours.

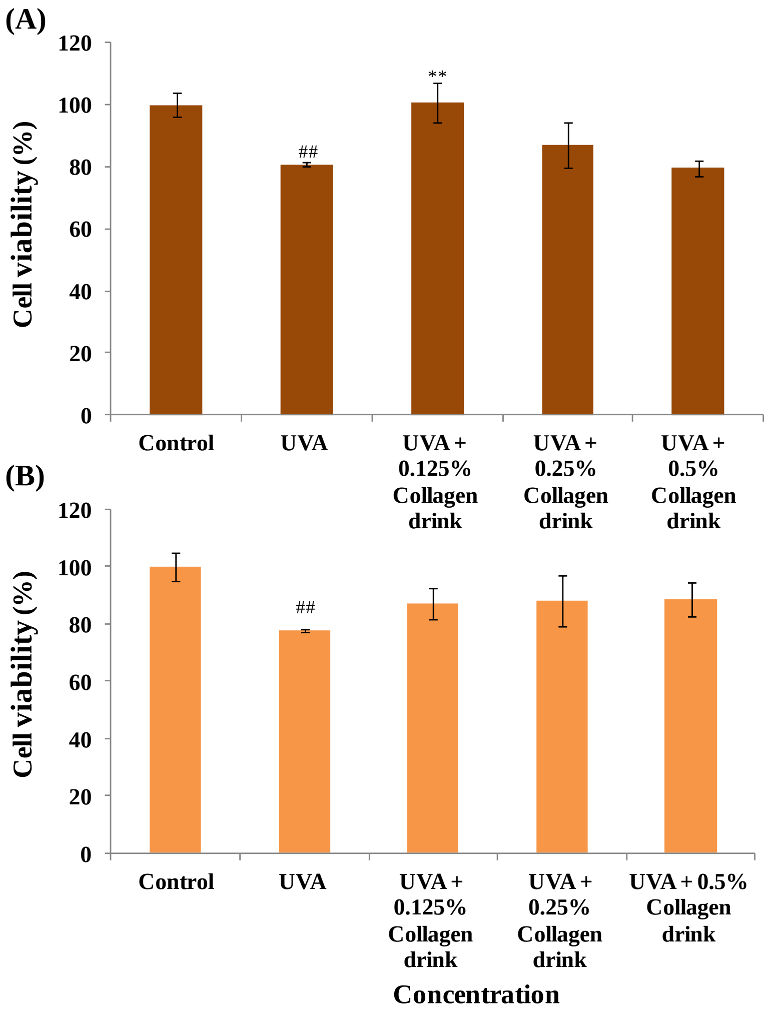

Figure 2. Result of cell viability of fibroblasts. (A) CCD-966Sk cells were treated with 0%, 0.125%, 0.25%, or 0.5% for 24 hrs following UVA treatment (10 J/cm^2). (B) CCD-966Sk cells in 0.125%, 0.25%, or 0.5% collagen drink were treated with UVA (10 J/cm^2) followed by 24-hour incubation. (n = 3, mean value ± S.D.) (Corresponding to the control group: $^{\#\#}$, p < 0.01; corresponding to the UVA group: **, p < 0.01)

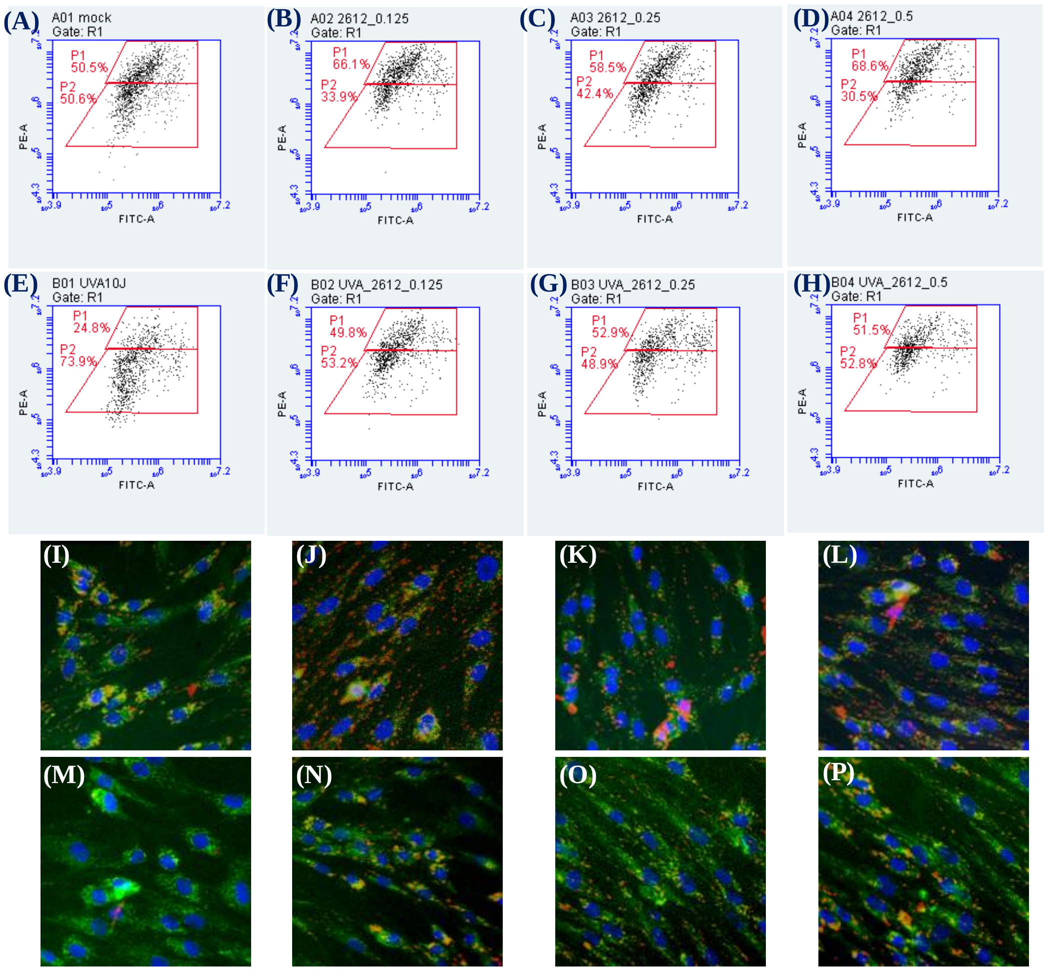

Figure 3. Results of flow cytometry pattern and fluorescence image of imitochondrial activity in fibroblasts. CCD-966Sk cells were treated with JC-1 dye (red signal) to assess the mitochondrial activity in fibroblasts and their signals were analysed by a flow cytometry by the percentage of cell signal at the P1 zone, which was defined by a ratio of 1(green signal from FITC channel) : 1 (red signal from PE-A channel). (A, I) Control. (B, J) 0.125% Collagne drink. (C, K) 0.25% Collagne drink. (D, L) 0.5% Collagne drink. (E, M) UVA only. (F, N) UVA + 0.125% Collagne drink. (G, O) UVA + 0.25% Collagne drink. (H, P) UVA + 0. 5% Collagne drink

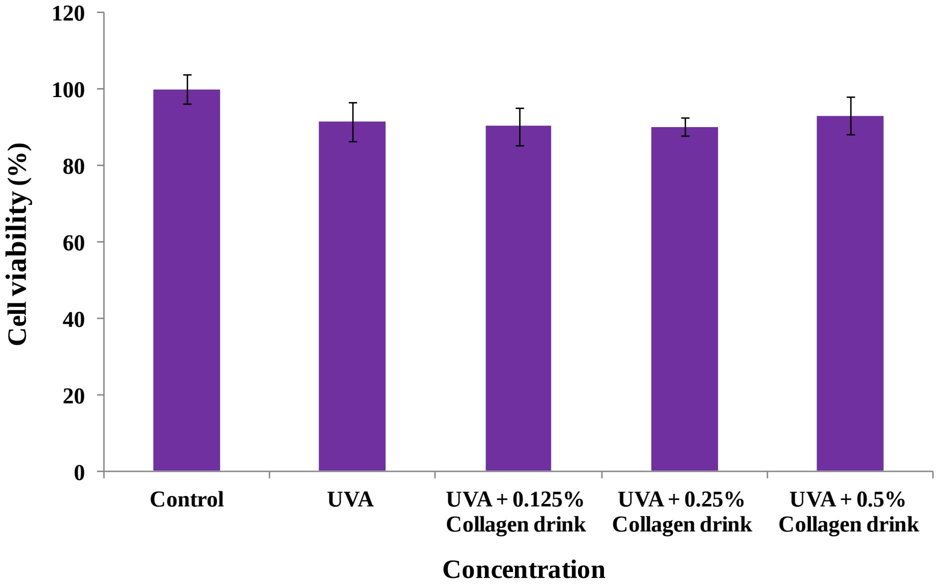

Figure 4. Result of cell viability of fibroblasts. CCD-966Sk cells were treated with 0.125%, 0.25%, or 0.5% for 48 hrs. (n = 3, mean value ± S.D.)
